# Syphilis and Veneral Diseases

**Published:** 1877-01

**Authors:** 


### VI. SYPHILIS AND VENEREAL DISEASES.

1. *Syphilis of Pregnant Women.* DARVOSKY. (*Memorabilien*, 1876, No. 10.)

Although, as a rule, the heroic treatment of diseases ought to be avoided in women far advanced in pregnancy, the writer holds that syphilis should be an exception to this rule. He advises the inunction cure, to be pursued energetically but judiciously, so that salivation be obviated, and that the simultaneous internal exhibition of potassic iodide be employed. One-half drachm of the mercurial ointment to be rubbed in daily, scrupulous cleanliness, frequent gargling with chlorate of potash, and thorough ventilation of the room. Such is the doctor's method, which he is not afraid to apply even during the last month of pregnancy.

2. *Communication of Syphilis by a Midwife.* DARVOSKY. (*Memorabilien*, 1876, No. 10.)

A young woman, married one year, had been delivered of a healthy child eight weeks before. She had been healthy during her pregnancy, and in her confinement nothing occurred to attract special attention. But shortly after that time she felt a burning and itching sensation about the genitals, which was soon followed by a tumefaction of the labia majora. The doctor found, on examination, broad condylomata of the labia and around the anus, roseola syphilitica, angina syphilitica, and indurated glands of the inguinal and cervical region. The husband had broad condylomata upon the penis and scrotum, but as yet no sign of universal lues; he admitted having had sexual intercourse with his wife five weeks after her confinement. The baby was perfectly healthy; the most scrupulous examinations could not detect anything suspicious. The woman showing the general signs while her husband had the initial symptoms of syphilis, she must have contracted the disease first. The doctor suspecting the midwife, found her affected with broad condylomata and induration of the glands; he further ascertained that she, after attending a syphilitic woman, had an ulcerated finger; that this ulcer was not entirely healed when she attended the above lady; and that another lady whom she had attended at about the same time had since then showed signs of syphilis.

3. *Suspected Vaccinal Syphilis.* GUERIN. (*L' Union Médicale*, November 23, 1876.)

Guérin presented to the Paris Academy of Medicine a little patient who had been vaccinated nine days after birth — seven weeks prior to date. The immediate results of the vaccination had been quite normal, the vesico-pustules becoming well developed. On the eighth day the virus from the latter had been employed in the vaccination of an elder brother, in whose case the evolution of the disease and the subsequent cicatrization had been entirely regular.

In the case of the first child, however, deep ulceration had occurred at the three sites of inoculation, with indurated edges, quite analogous to that found in indurated chancre; but the corresponding lymphatic ganglia were not engorged. On the same arm, however, was a perfectly cicatrized periostosis. The difficulty was pointed out of establishing the influence of syphilis in a case which displayed tertiary symptoms after a few weeks, without the occurrence of symptoms intermediate between it and the primary lesion. The reporter consequently assumed the phenomena to be manifestations of the strumous diathesis in an infant vaccinated very early after birth; others who observed the child being inclined to adopt the same opinion.

4. *Communication of Syphilis by Milk.* VOSS. (*Petersburg Med. Wochenschrift*, No. 23, 1876.)

Three prostitutes were inoculated with the milk of a woman affected with papular syphilis, who suffered also from moist mucous papules of the anal and genital regions — the mammary glands being free from disease. A syringeful of milk, expressed from one breast, was injected into the tissues of each prostitute by means of a Pravaz syringe. One who had been previously syphilitic, suffered no inconvenience; the second had urethritis, and was not affected; the third was a young girl sixteen years old, free from syphilis, who was injected on the eleventh day after her admission to the hospital. The inflammation and local suppuration excited, subsided in one week; but forty days after the inoculation papules were developed around the site of the injection, and in five days a maculo-papular syphilide appeared over the body, with concomitant adenopathy — these symptoms disappearing after the employment of mercurial inunction.

5. *The Tincture of Tayuya in Syphilis.* Longhi. (*Gazz. Med. Italiana-Lombardia*, November 25, 1876.)

The third observation of the author concerned the case of a child three months old, who had been suckled by a nurse after the latter had been infected with syphilis by a former nursling. The infant exhibited over the groins and external genitalia, ten or twelve papules, of the size of large peas, rounded in contour, and covered with a whitish offensive secretion — these lesions being implanted upon an integument which was considerably reddened. Upon the lips of the mouth were several small whitish ulcers with reddened borders. Similar sores were also found upon the tongue. The diagnosis was made of papular syphilis of the genitals and mouth. Three teaspoonfuls daily were given of a solution containing one hundred and fifty drops of the dilute tincture of tayuya (Ubicini Brothers') in eight hundred grammes of water. The parts were bathed three or four times daily in the same solution. In a fortnight the cure was complete, the author expressing a regret that he could not have tested the treatment further, and made continued observation of the case.

The fourth case of the series is that of a patient thirty-six years old, who had had repeated attacks of gonorrhœa, and a gleety discharge for fifteen years. During this period he had suffered frequently from articular and muscular pains, which were accompanied by swellings upon the manubrium of the sternum, the occipital and parietal bones, the dorsal vertebræ and ribs. There had been also copper-colored blotches of the surface — one upon the left pectoral region being as large as the hand of an adult. He had vainly tried many kinds of empirical treatment, not neglecting the use of mercury, iodine, hot baths, etc. On the first of September he could hardly move in bed; the lateral movements of the neck were evidently accomplished only with difficulty, and all sudden motions (sneezing, coughing, etc.,) were excessively painful. The bones were enlarged, as described above; while the eruption and gleety discharge observed did not seem of recent development. A diagnosis was established of macular syphilide, with syphilitic periostitis and arthritis. For several preceding months the patient had taken no medicine; but in July he had been subjected to a hydrotherapy, which left him in a more unfavorable condition than at first. On the 12th of September the treatment by tayuya was begun, in doses of fifteen drops of the dilute tincture in two hundred grammes of water. In five days the patient could tolerate forty drops in the same quantity, which were continued till September 20th. On that day he took eighteen drops of the mother tincture, increased to forty of the same by October 1st, then slight abdominal pain was complained of, when but thirty-five drops of the strong tincture were ordered to be employed. The patient had now for several days stated that his pains were not one-fifth of what they were formerly; the osseous enlargements had well nigh disappeared, and also the cutaneous maculæ, with the exception of that upon the breast, which was smaller by two-thirds than at the beginning of treatment; the gleet had completely disappeared. Some recurrence of pain occurred after drinking half a litre of wine with his companions, when the dose was again increased to forty drops. On October 5th, the commercial tincture was substituted for that employed, in eighty drops daily (the mother tincture being exhausted), and the latter dose gradually reduced to fifty drops by November 10th, the date of the writer's last observation. All the osseous enlargements have disappeared; but the pectoral macule is still visible, and the pain only appears for a day after drinking wine, becoming constipated, or on exposure to the cold.

6. *The Hypodermic Treatment of Syphilis.* NEUMANN. [Memoir presented to the Imperial Academy of Medicine, Vienna.] (*Anales de Ciencias Méd.*, November 20.)

After a brief historical sketch of this mode of treatment, Neumann proceeded to show the value of injections made with the albuminate of mercury. The advantages claimed were, that there is the least degree of reaction, and no abscesses nor gangrene follow. Under any circumstances before absorption of mercury can occur, it must be united with albuminous substances — precisely what occurs when the mineral is taken into the stomach. Bamberger's solution (of the albuminate of mercury), however, contains the sublimate already combined with albuminoid substance, and hence it enters the tissues of the body without requiring further transformation. It is thus readily absorbed, and the tissues of the body are not altered by its admission. Therefore it is especially useful when speedy effects are desired, as its operation is more rapid than that of the protiodid and corrosive sublimate when administered by the mouth. Besides, the number of injections can be readily regulated, the dosage is completely under the control of the operator, and the stomach and bowels are left undisturbed and unirritated. Stomatitis also is rare. The disadvantages which have been claimed by certain authors, viz.: elevation of the temperature, increase in the frequency of the pulse, colliquative diarrhœa, formation of abscesses, etc., have not resulted, in Neumann's experience, from the employment of Bamberger's solution.

